# An End-to-End Artificial Intelligence of Things (AIoT) Solution for Protecting Pipeline Easements against External Interference—An Australian Use-Case

**DOI:** 10.3390/s24092799

**Published:** 2024-04-27

**Authors:** Umair Iqbal, Johan Barthelemy, Guillaume Michal

**Affiliations:** 1 SMART Infrastructure Facility, University of Wollongong, Wollongong, NSW 2500, Australia; 2 APA Group, Sydney, NSW 2000, Australia

**Keywords:** edge computing, object detection, pipelines, easement activities, critical infrastructure monitoring, AIoT

## Abstract

High-pressure pipelines are critical for transporting hazardous materials over long distances, but they face threats from third-party interference activities. Preventive measures are implemented, but interference accidents can still occur, making the need for high-quality detection strategies vital. This paper proposes an end-to-end Artificial Intelligence of Things (AIoT) solution to detect potential interference threats in real time. The solution involves developing a smart visual sensor capable of processing images using state-of-the-art computer vision algorithms and transmitting alerts to pipeline operators in real time. The system’s core is based on the object-detection model (e.g., You Only Look Once version 4 (YOLOv4) and DETR with Improved deNoising anchOr boxes (DINO)), trained on a custom Pipeline Visual Threat Assessment (Pipe-VisTA) dataset. Among the trained models, DINO was able to achieve the best Mean Average Precision (mAP) of 71.2% for the unseen test dataset. However, for the deployment on a limited computational-ability edge computer (i.e., the NVIDIA Jetson Nano), the simpler and TensorRT-optimized YOLOv4 model was used, which achieved a mAP of 61.8% for the test dataset. The developed AIoT device captures the image using a camera, processes on the edge using the trained YOLOv4 model to detect the potential threat, transmits the threat alert to a Fleet Portal via LoRaWAN, and hosts the alert on a dashboard via a satellite network. The device has been fully tested in the field to ensure its functionality prior to deployment for the SEA Gas use-case. The AIoT smart solution has been deployed across the 10km stretch of the SEA Gas pipeline across the Murray Bridge section. In total, 48 AIoT devices and three Fleet Portals are installed to ensure the line-of-sight communication between the devices and portals.

## 1. Introduction

High-pressure pipelines are a critical component of the infrastructure for transporting oil, gas, and other hazardous materials over long distances [1,2,3,4]. Generally, the network of pipelines passes through densely populated areas, sensitive environmental regions, and high-seismic-activity regions, which increase the likelihood of interference threats leading to potential accidents [2,4]. A few common third-party interference activities that may be a threat to high-pressure pipelines include excavation (e.g., digging, trenching, drilling), construction activities (e.g., roads, bridges, buildings), agricultural activities (e.g., ploughing, land clearing, irrigation installment), and utility installments (e.g., installing communication cables) [2,5,6]. These activities may result in damaging the pipelines, which can have severe consequences such as safety hazards (e.g., fire, explosion), environmental risks (e.g., soil/water contamination, damage to wildlife), and operational disruptions (e.g., service downtime, loss of revenue) [7,8]. Therefore, the proactive management of these threats is essential to ensure that inference accidents do not occur.

Under AS 2885 [9], pipeline licensees are required to complete a Safety Management Study (SMS) to demonstrate that adequate physical and procedural measures are in place to protect the pipeline [9]. In this context, there are several measures taken to avoid interference accidents and to ensure the safe operations of the pipeline including pipeline location marking (e.g., above-ground markers and signs) [10], regular visual and internal inspections (e.g., on-ground patrolling, drones, smart pigs) [11], training and education programs (e.g., awareness workshops, trainings) [12], coordination with local authorities (e.g., work plans, information sharing), and implementing safety measures (e.g., fencing, signage) [13]. Despite the implementation of so many preventive measures, interference accidents can still occur for several reasons including human error, equipment failure, lack of awareness, extreme weather, and malicious activities [4,14]. Conventional methods of periodic patrolling to detect third-party activity near pipelines are restricted in their effectiveness due to the limited time-frame in which threats can be detected. As population growth and urban expansion lead to increased easement activity near pipelines, the limitations of current patrolling methods become more pronounced [15]. These limitations are not unique to Australia and are likely to be a common issue in the global pipeline and linear infrastructure industry.

Therefore, the need for high-quality detection strategies is vital for the few occasions where preventative controls fail to avoid potentially catastrophic consequences. Satellite photogrammetry and drone-based solutions are one potential approach; however, these come with several operational limitations. Satellite-photogrammetry-based solutions are highly impacted by the weather conditions, and it expensive to obtain high-resolution images and in real time. The drone-based solution may work at the local scale; however, they are limited to short operational times. Drones may be used for on-demand inspections; however, this is not a viable solution for continuous monitoring. Artificial Intelligence, computer vision, edge computing, and Artificial Intelligence of Things (AIoT) technologies have taken off in the recent few years and are efficiently addressing many surveillance-/detection-related real-world problems (e.g., pedestrian detection [16], wildlife monitoring [17], culvert blockage detection [18], plastic bag contamination detection [19]). Detecting pipeline easements against external interference using computer vision poses a unique challenge with no existing literature or visual dataset. However, several studies have explored computer vision techniques for construction machinery detection, which is highly relevant to this problem. In 2018, Kim et al. [20] used an R-FCN object-detection model in the context of detecting construction equipment in a dataset consisting of around 3000 images extracted from the ImageNet dataset. The trained model was able to achieve a mAP of 95% for the excavator class. Later, in 2019, Robert and Golparvar-Fard [21] used convolutional neural network (CNN)-based object-detection (i.e., RetinaNet) and -tracking algorithms for the activity analysis of earthmoving equipment. These analyses were based on only 10 videos captured at very specific construction sites at the ground level. For this custom dataset, a detection AP of 97% was reported for the excavator class. Although a high performance was reported, the model was trained for two classes (excavators, dump trucks), making it a relatively easier problem in comparison to the presented 13-class problem. In a recent research work, Xiao and Kang [22] used the You Only Look Once version 3 (YOLOv3) object-detection model with Simple Online and Real-time Tracking (SORT) to track construction machines. A dataset of around 10,000 images named the Alberta Construction Image Dataset (ACID) [23] of 10 types of construction machines with excavators as one of the classes was used for training the object-detection model. The authors did not report the detection model’s accuracy for each separate from the tracking. Despite not directly addressing pipeline easement detection, these studies highlight the potential of object-detection models in similar contexts, suggesting the promise of addressing this challenge from a computer vision perspective. Notably, none of the literature works discussed deployment on edge computing hardware in the field, which is crucial for an end-to-end solution.

This paper proposes to develop an end-to-end AIoT solution to alert the pipeline operator of a potential interference threat in real time. The idea presented is to develop a smart visual sensor capable of processing the live video feed from a local camera, detect the potential interference threat using a state-of-the-art computer vision algorithm, and send the alert (i.e., a message notifying the operator about the threat and containing the information about the threat type, time stamp, and device_id) to the pipeline operator in real time to avoid an accident. The development of the AI system to automatically detect the interference threat is the core of this solution and is based on the object-detection model. For this research, two object-detection models including YOLOv4 and DETR with Improved deNoising anchOr boxes (DINO) were implemented and compared. To train the models, a custom dataset (i.e., Pipeline Visual Threat Assessment (Pipe-VisTA)) was developed from multiple resources and annotated for training. The anticipated contributions are as follows:

1. The development of the custom Pipe-VisTA dataset consisting of 10,181 images to facilitate the training of the computer vision object-detection model.
2. The development of an end-to-end interference-threat-detection solution by designing a smart visual sensor based on AIoT capable of processing the live video from a camera using an edge computer equipped with the trained object-detection model (e.g., YOLOv4, DINO) to detect the potential interference threat and transmitting the alert message to the pipeline operator using LoRaWAN in real time to avoid an accident.
3. The installment and field validation of the developed solution for the SEA Gas use-case to assess the actual real-world performance of the proposed system.

## 2. Pipeline Visual Threat Assessment (Pipe-VisTA) Dataset

A custom dataset (i.e., Pipe-VisTA (Pipe-VisTA is not publicly available and a propriety dataset. However, a public version of it may be provided upon request.)) was established to train the computer vision models for automated inference threat detection using multiple sources including images from the web and videos of relevant excavation/earthmoving equipment. In total, approximately 5000 raw images and 15 h of relevant videos were collected to establish the dataset. The final dataset consisted of 13 classes covering most of the potential interference objects (see Table 1 for the classes). All the images in the final dataset were in JPG format with the RGB color scheme and resized to 1024 × 768 pixels. The dataset offered challenges in terms of multiple threats present in the single image.

**Figure 1:**
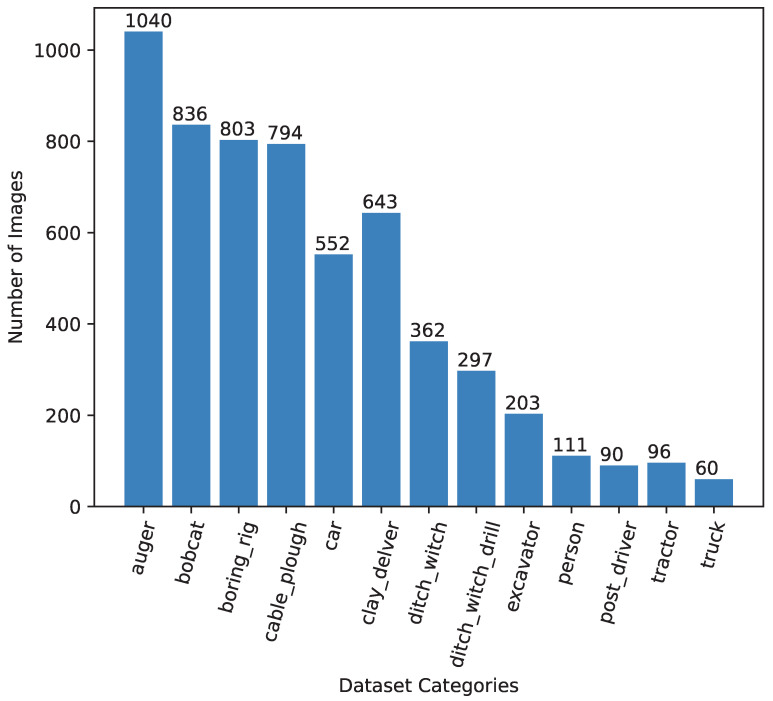
Distribution of Pipe-VisTA across different categories.

**Figure 2:**
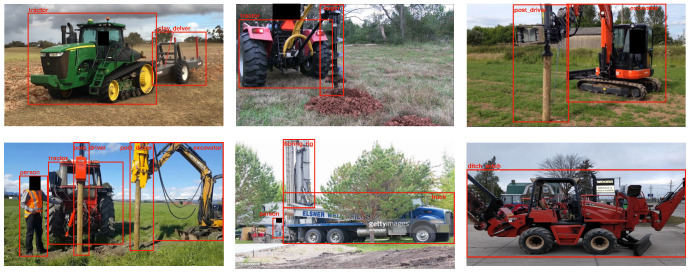
A few annotated samples from the Pipe-VisTA dataset.

As a labeling schema, within the images, objects belonging to each class in Table 1 were annotated using bounding boxes to facilitate the object-detection model’s training. Annotations were created using the *LabelBox* [24] tool, and labels were exported in KITTI format for training the computer vision model. The final dataset consisted of 10,181 images (i.e., 7127 (70%) for training, 2036 (20%) for validation, 1018 (10%) for testing) with a total of 45,811 bbox annotations covering all 13 categories. Figure 1 shows the distribution of the annotated instances for each class and clearly demonstrates that auger, person, boring_rig, tractor, post_driver, and truck were the dominant classes with more than 500 annotated instances. Pipe-VisTA is a novel dataset presented as a benchmark in this research to facilitate the development of computer vision solutions for interference threat detection. Figure 2 shows some annotated samples from Pipe-VisTA.

## 3. The Proposed AIoT Smart Sensing Solution

**Figure 3:**
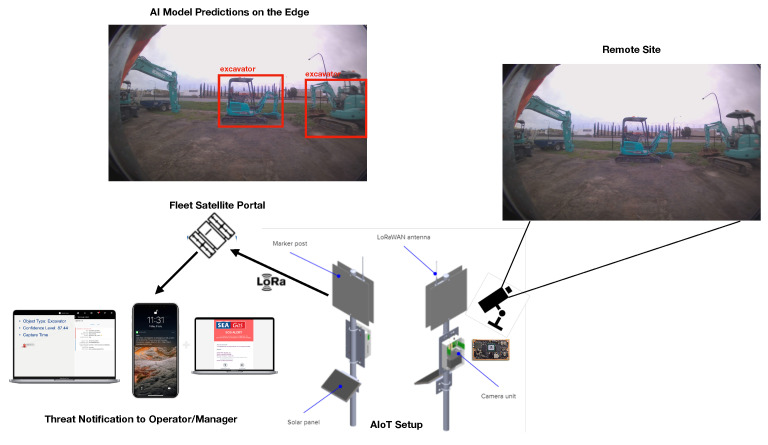
Visual illustration of the proposed AIoT solution for external interference threat detections.

An AIoT smart sensing solution has been proposed and developed to detect the external interference threat and alert the operator to avoid potential accidents. The proposed solution aims to monitor the remote site using a camera (see Table 2 for the NANO-H136 camera’s specifications by Leopard Imaging Fremont, CA, USA), detect the external interference threat using the AI model (i.e., YOLOv4, DINO) on the edge computing device, and transmit the alert information (i.e., type of threat, time) using the LoRaWAN gateway [25] to a dashboard accessible by the operator. Figure 3 shows the visualization of the proposed solution. Given the utility of the solution at remote sites, the edge computing paradigm is followed in which images/data are processed on the device and only the relevant results are transmitted. The low network bandwidth requirement is one of the main advantages of the edge computing approach, which makes it suitable for Low-Power Wide-Area (LPWAN) networks such as LoRaWAN (i.e., a protocol offering long-range communication at a low bit rate and low power consumption).

The AIoT smart sensing device and solution consists of the following core components (as identified in Figure 3):

- A standard web camera to capture the remote site images to be processed by the AI model for potential threat detection.
- An NVIDIA Jetson Nano 4G edge computer (i.e., ARM-based embedded device to accelerate the AI computations) equipped with the trained AI model (e.g., YOLOv4, DINO) to detect external interference threats.
- A radio module to handle the wireless LoRaWAN communications and transmit the results of the AI model.
- A solar-based battery system to power the AIoT sensing device in remote areas.
- A Fleet Portal to transmit the alert via Fleet’s satellite network, which enables connectivity between the cloud and terrestrial network elements and enables coverage in areas with no other connectivity options.
- A dashboard to host the transmitted alert(s) accessible by the operators and managers to be informed in a timely manner about any potential threat. The alters reach Nebula from the Fleet network, the control platform where the data are aggregated and enabling all network management operations to be performed.

As the operational flow, the AIoT device iterates through the following steps every fifteen minutes:

1. Turn the power on, and wake up the device.
2. Acquire the image from the camera
3. Process the image using the trained AI model on the edge computer to identify the potential external interference threat.
4. If a threat is detected, transmit the alert (i.e., type of threat, timestamp, device_id) to the Fleet Portal using the LoRaWAN protocol.
5. Transit to hibernation mode to save power.

## 4. Training and Evaluation of Threat-Detection AI Model

This section presents the details about the training and evaluation of the object-detection models (i.e., YOLOv4, DINO) for the external interference threat detection. The section is divided into subsections providing a brief theoretical overview of the implemented object-detection models, details about the protocols adopted to train the models, performance evaluation measures, training results, and testing results.

### 4.1. Theoretical Background to Object-Detection Models

#### 4.1.1. You Only Look Once Version 4 (YOLOv4)

YOLOv4 is a state-of-the-art object-detection model that uses a deep convolutional neural network to detect and localize objects in an input image [26]. It belongs to the family of deep learning models that offers a good equilibrium between speed and accuracy. Indeed, it can offer real-time performance when deployed on systems with limited computational resources while still delivering top accuracy. The architecture of the YOLOv4 model is designed to be able to detect objects at different scales (i.e., small, medium, large) to compensate the object’s perceived size in an image based on its distance to the camera. The YOLOv4 model consists of a backbone network, a neck network, and a head network. The backbone network is based on the CSPDarknet53 architecture, which uses a Cross-Stage Partial (CSP) network module to improve the flow of information across different layers of the network. The neck network uses a Spatial Pyramid Pooling (SPP) module to allow the model to detect objects of different sizes. The head network uses a YOLOv3-style detection head to predict bounding boxes and object classes.

The YOLOv4 model works by processing an input image in three grids (one for each detection scale) through the backbone network, neck network, and head network to generate a set of bounding boxes and object classes. The bounding boxes are represented as four coordinates (x,y,w,h), where (x,y) is the center of the box, *w* is the width of the box, and *h* is the height of the box. The object classes are represented as a probability distribution over a set of predefined classes. The output of the YOLOv4 model can be represented as a set of bounding boxes and corresponding object classes (see Equation (1)).

(1) $$Y={\left(x_{i},y_{i},w_{i},h_{i},c_{i}\right)}_{i=1}^{N},$$

where *N* is the number of detected objects, $x_{i}$, $y_{i}$, $w_{i}$, and $h_{i}$ are the coordinates of the *i*-th bounding box, and $c_{i}$ is the probability distribution over the set of predefined classes for the *i*-th object.

The YOLOv4 model is trained using a combination of a classification loss and a localization loss. The classification loss measures the difference between the predicted class probabilities and the true class probabilities, while the localization loss measures the difference between the predicted bounding boxes and the ground truth bounding boxes. The total loss is a weighted sum of the classification loss and the localization loss (see Equation (2)).

(2) $$L=\lambda_{cls}L_{cls}+\lambda_{loc}L_{loc},$$

where $L_{cls}$ is the classification loss, $L_{loc}$ is the localization loss, and $\lambda_{cls}$ and $\lambda_{loc}$ are hyperparameters that control the relative importance of the two losses.

The localization loss is calculated using the mean-squared error (MSE) between the predicted bounding boxes and the ground truth bounding boxes (see Equation (3)).

(3) $$L_{loc}=\frac{1}{N_{obj}}\sum_{i=1}^{N_{obj}}\sum_{j\in x,y,w,h}\lambda_{j}\cdot \mathrm{MSE}\left(y_{i}^{j},{\hat{y}}_{i}^{j}\right),$$

where $N_{obj}$ is the number of objects in the image, $y_{i}$ and ${\hat{y}}_{i}$ are the ground truth and predicted values of the *i*-th bounding box, $\lambda_{j}$ is a hyperparameter that controls the relative importance of the *j*-th coordinate, and $\mathrm{MSE}\left(a,b\right)={\left(a-b\right)}^{2}$ is the mean-squared error between *a* and *b*.

To remove duplicate detections of the same object, the Non-Maximal Suppression (NMS) technique is applied. If there is an Intersection over Union (IoU) between two bounding boxes $B_{m}$ and $B_{n}$ greater than a predefined NMS threshold γ, i.e., if IoU$(B_{m},B_{n})>\gamma$, then the bounding box with the least object confidence score is removed.

#### 4.1.2. DETR with Improved deNoising anchOr Boxes (DINO)

DINO introduces a groundbreaking approach to end-to-end object detection, surpassing previous DETR-like models in both performance and efficiency [27]. At the core of DINO’s architecture are several novel techniques designed to enhance training effectiveness and prediction accuracy. One key innovation is the contrastive denoising training method, which aims to minimize a composite loss function consisting of various reconstruction losses (such as the l1 and GIOU losses for box regression) and focal losses for classification. This loss function is formulated to penalize false positives and encourage the model to distinguish between useful anchor boxes and noise. By incorporating positive and negative samples of ground truth boxes with different noise scales, DINO effectively learns to reject useless anchors and make more accurate predictions.

Another crucial aspect of DINO’s architecture is the mixed query selection strategy, which optimizes the initialization of anchor boxes while maintaining learnable content queries. This strategy is mathematically represented by the set of queries $Q=\{q_{1},q_{2},\cdots ,q_{n}\}$, where *Q* denotes the set of queries and $q_{i}$ represents the *i*-th query. By selecting initial anchor boxes as positional queries from the output of the encoder and keeping content queries learnable, DINO leverages spatial priors while still allowing flexibility in learning content features. This approach enhances the model’s ability to capture spatial relationships and improve prediction accuracy.

**Figure 4:**
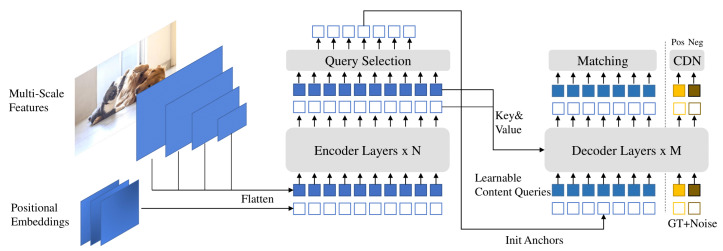
Architecture of the DINO model [28].

Furthermore, DINO incorporates a novel look forward twice scheme for box prediction, which iteratively refines the initial boxes and predicted offsets to improve prediction quality. This scheme involves updating the parameters based on both the current and future layers, as represented by the equation $\Delta b_{i}={\mathrm{Layer}}_{i}\left(b_{i-1}\right)$, where $b^{\prime }=\mathrm{Update}\left(b_{i-1},\Delta b_{i}\right)$, $b_{i}^{\prime }$ represents the refined box, $b_{i-1}$ denotes the initial box, and $\Delta b_{i}$ denotes the predicted offset. By iteratively improving both the initial boxes and the predicted offsets, DINO achieves more accurate and reliable predictions, particularly in scenarios with complex spatial relationships or small objects. Figure 4 shows the architecture of the DINO model.

### 4.2. Training Protocols and Evaluation Measures

The YOLOv4 and DINO models were trained using the NVIDIA *Train Adapt Optimize* (*TAO*) version 3.0 framework, which is natively based on *Tensorflow* and *PyTorch*. For the YOLOv4 model, the CSPDarkNet53 network pretrained on the Google OpenImages dataset [29] with 49,468,412 trainable parameters was used as the backbone architecture. For the training, the number of bounding boxes per cell was set to three, the object confidence threshold was set to 0.01, and the NMS clustering threshold was set 0.5. It was trained for 1000 epochs to ensure convergence. The DINO model was trained with the FAN_Tiny backbone for 200 epochs. For all the model training, the transfer learning approach was adopted to avoid the requirement of a huge dataset, large computational resources, and long training times for training the model from scratch. All the models were trained using a desktop machine equipped with two NVIDIA T4 Graphical Processing Units (GPUs) with 16 GB of memory each. The main parameters for the training process are summarized in Table 3. The model hyperparameters were manually tuned to achieve the best results.

The trained model was evaluated using validation and test datasets with images unseen during the training process. The split of train–val–test (i.e., 70–20–10) was used to ensure that the model was not overfitting. As an evaluation metric, the Mean Average Precision (mAP) was used, which is based on the IoU (i.e., a measure of the overlap between the predicted bounding box and the actual bounding box (“ground truth”) created during the annotation of the dataset). A prediction by the AI is deemed correct if the IoU between the predicted bounding box and the ground truth is above a given threshold *t*. The mAP is a metric in [0,1] summarizing the performance of the AI for different overlapping thresholds *t*. A value close to 1 indicates an accurate model.

### 4.3. Results

**Figure 5:**
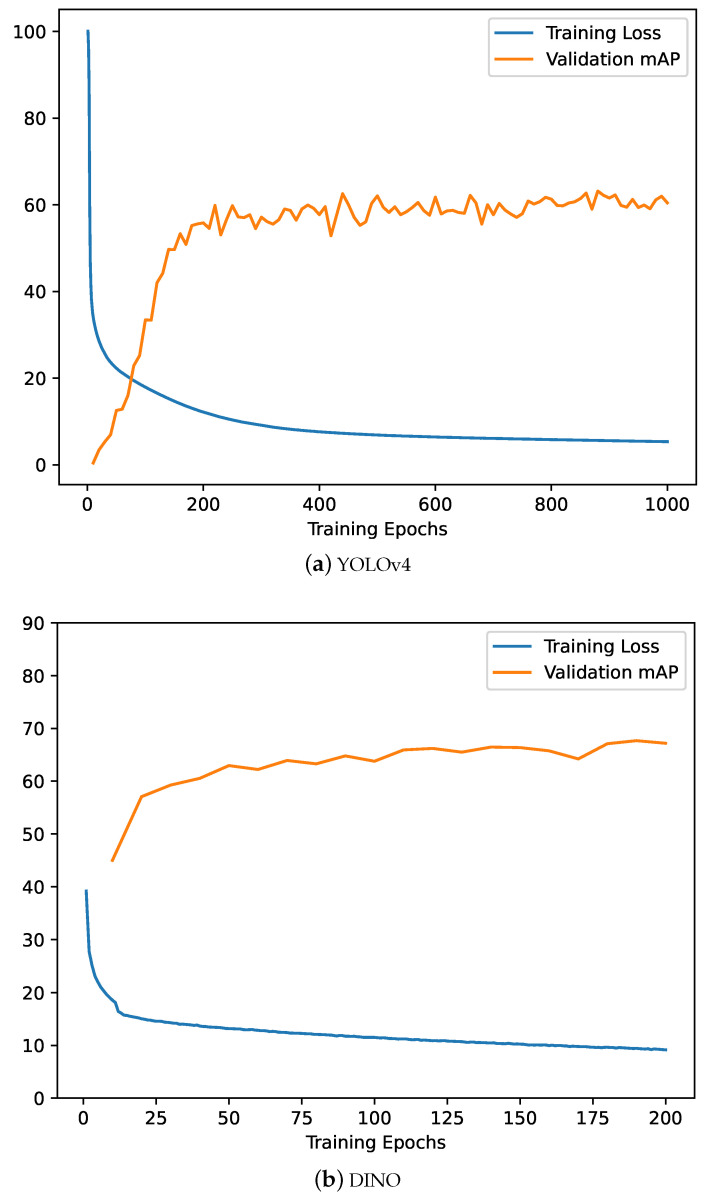
Training loss and validation mAP curves for the trained object-detection models.

The training and validation results for the object-detection models (i.e., YOLOv4, DINO) to detect the external interference threat are reported quantitatively and graphically. The training loss and validation mAP curves against the number of epochs for each model are presented in Figure 5. For both models, the training loss follows the negative exponential curve (i.e., the loss is decreasing as the number of epochs increases), which suggests a normal training process to learn the detection task. Similarly, the validation mAP follows the positive exponential trend (i.e., the mAP increases as the number of epochs increases), indicating that there is no overfitting during the training process. For the YOLOv4 model, the training loss was reported to be settled at 5.3, while the validation mAP was reported to be 0.631 after 1000 epochs. For DINO, the training loss converged at 9.15, while the validation mAP settled at 0.671 at the end of 200 epochs.

**Figure 6:**
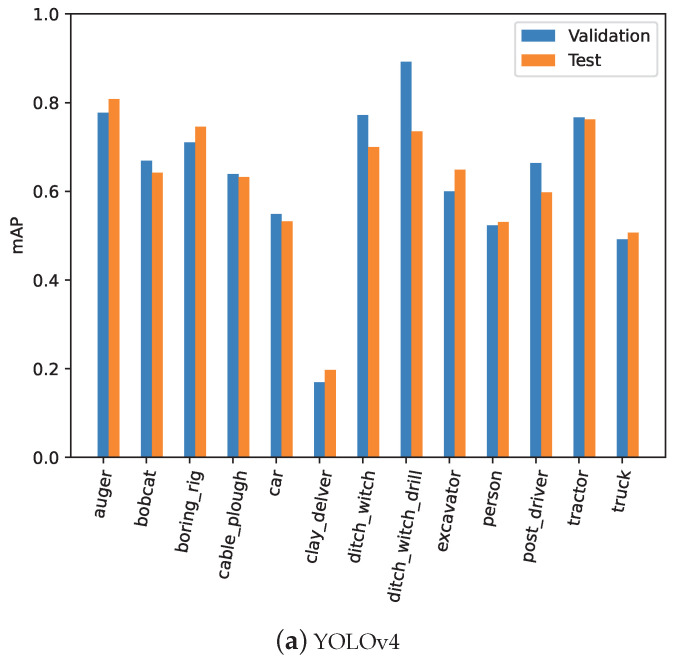

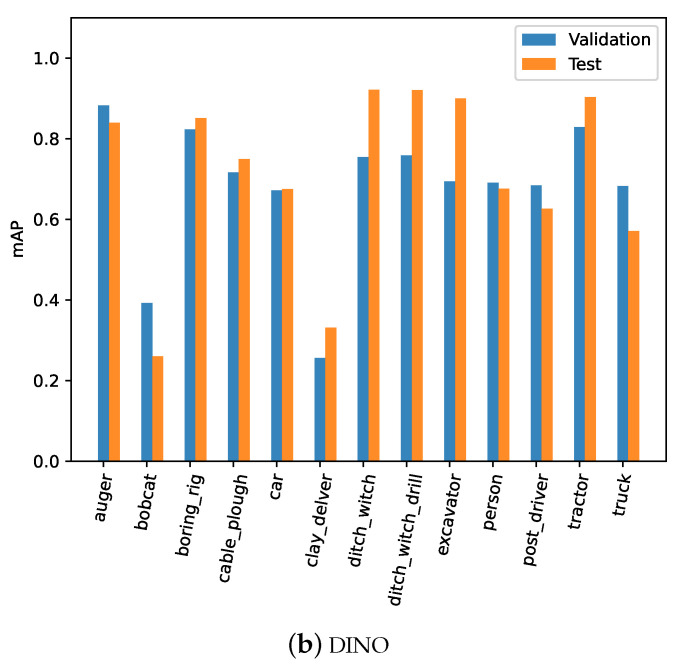
Comparison of the validation and test mAPs for the trained object-detection models.

In addition to the validation performance, the trained models were also evaluated against an unseen test dataset, and the results were compared with the validation to ensure that the models learned generalized features and no overfitting occurred during the training process. Table 4 presents the detailed categorywise quantitative results for the validation and test mAP for the last training epoch models. The results indicate that the validation and test performances of the trained models were relative to each other, demonstrating generalized training. DINO emerged as the best model among all with a validation mAP of 0.671 and a test mAP of 0.712. In terms of separate categories, the best validation mAP was observed for the tractor (i.e., 0.829), boring_rig (i.e., 0.823), and auger (i.e., 0.822) categories. On the other hand, the lowest mAP was observed for the clay_delver (i.e., 0.256) and bobcat (i.e., 0.392). One potential reason for the lower mAPs for clay_delver could be associated with images related to these classes in the dataset. Enhancing the dataset with more images related to these categories in the future will potentially increase the performance. Although the superior performance was achieved by the DINO model, given the requirement of deploying a solution on a limited-computational-power edge computer (i.e., NVIDIA Jetson Nano), a simpler model of YOLOv4 optimized with TensorRT for deployment was selected. The YOLOv4 model achieved a validation mAP of 0.631 and a test mAP of 0.618. Figure 6 graphically compares the categorywise mAPs for the validation and test datasets.

**Figure 7:**
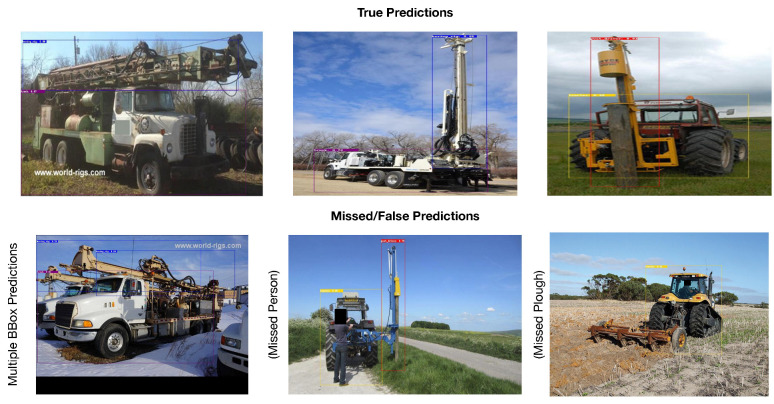
A few selected true and false predictions by the trained YOLOv4 model.

Figure 7 presents a few selected images from the test dataset, where the YOLOv4 model was able to correctly predict the threat type and where the model failed or had missing predictions. It can be observed that the model was able to accurately predict for the first row of images with the boring rig, truck, tractor, and post driver categories. In terms of false/missing predictions, the model resulted in multiple bbox predictions for the same object, which is a common issue and can be resolved by simply picking the best scoring bbox and rejecting the others. Further, the model, sometimes, partially detected the objects in the image and missed the other object, for example, in Figure 7, “tractor” and “post driver” were accurately predicted, while the “person” category was missed. This observation suggests adding more images related to missed categories in the training dataset.

## 5. Field Testing and Validation

**Figure 8:**
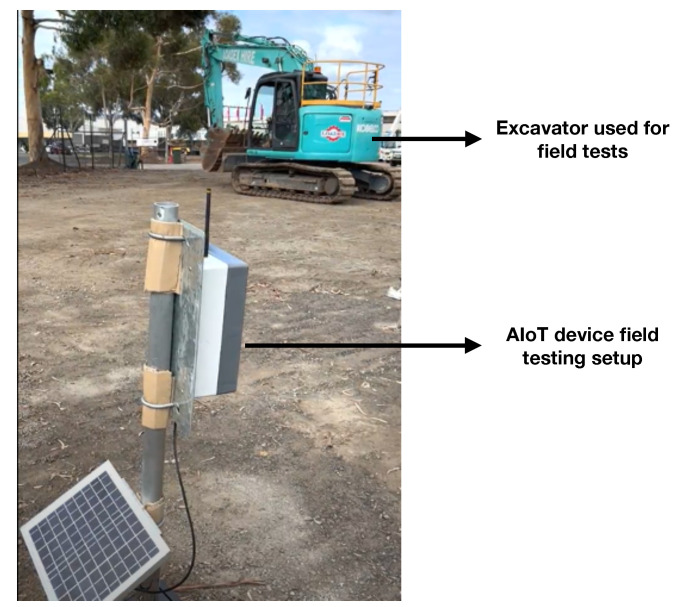
Field testing and validation setup for the AIoT device.

The developed AIoT device was tested and validated in the field with a controlled setup prior to scale deployment. The solution was tested for both hardware and software including the functionality and performance. One AIoT device was set up in the field with an excavator as the external interference to assess the performance (see Figure 8 for the field setup). Overall, the testing and validation included the assessment of the detection performance of the AI model for detecting a stationary and a moving excavator, testing the performance at different times of the day, testing the end-to-end data transmission, testing the hibernation mechanism, and determining the true positive, false positive, and false negative rates for the AI model.

### 5.1. Hardware Testing

Hardware testing of the AIoT device included the assessment of the data transmission, power management, resistance to water, and hardware health. A summary of each of the hardware tests is listed as follows:

- The alerts were successfully generated by the AIoT device during the field tests and went through a Portal, forwarding the data and alerts to the user interface. The LoRaWAN data transmission was reliable for the tests.
- In order for the AIoT device to remain operational when powered using batteries (12 V, 12 Ah) and the solar panel (10 W), the device was configured to be in hibernation mode and to wake up every 15 min to monitor the area in its field of view. The wakeup, detection, and hibernation operational flow for the device was successfully validated during the tests.
- The AIoT device was designed to be waterproof; however, minor condensation appeared on rare occasions specifically on days with large temperature variations.
- The temperature, humidity, and battery life sensors of the AIoT device were regularly transmitted via LoRaWAN messages to monitor the device health. The payload of the message contains the temperature in Celsius, the relative humidity in percentage, and the battery voltage. The field tests reported no unusual behavior from the sensors.

### 5.2. AI Model’s Performance

The tests related to the performance of the AI were divided into two parts: functional tests and a 24 h uninterrupted test. The functional tests involved detecting objects being stationary or in motion at different locations (distance and angle from the direct line of sight). Firstly, the results of the distance and angle tests imply that the camera struggled with objects at a long distance and distortions near the edge of the frames. This is likely due to the wide field of view of the camera lens, generating optical distortion and chromatic aberrations. Finally, the movement tests aimed to simulate the scenario of an excavator moving and with its arm ready to dig. With the arm automated and moving up and down, the unit can detect it successfully.

In the second testing phase, the unit was set up to run continuously for 24 h to collect data for false/true positive/negative analysis and luminescence tests. The luminescence tests were conducted to assess the daily operational time window when the unit is able to perform object recognition. Since the camera in the unit does not have night vision capability, the performance of the AI dropped after the camera went into the dark environment (between 21:00 and 06:00) during the test. The device was still capable of detecting objects at dusk, at dawn, and in direct sunlight scenarios. It should be noted that the AIoT device was programmed to only forward the highest confidence level for the same object detected over the three consecutive images taken every 15 min by the device. For example, if the device detects an excavator in the sequence of images with confidence levels of 90%, 91%, and 95% in images 1, 2, and 3, the unit will only forward the alert with the confidence level of 95%.

**Figure 9:**
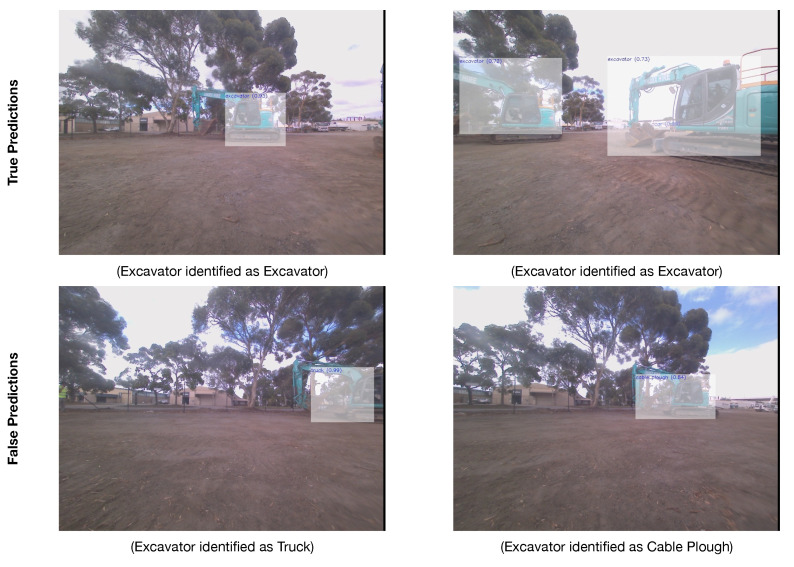
True and false predictions of the AI model in the field tests.

When considering all the categories that can be recognized by the AI, the unit achieved a true positive rate (detecting the right object) of 67.30%, a false positive rate (detecting the wrong object or missing a detection) of 32.70%, a true negative rate (not detecting an object when there is none) of 47.37%, and false positive rate (not detecting an object when there is one) of 52.63%. When only focusing the tests on the excavator category, the test results in the 24 h test had a false negative rate of 14.74% and a true negative rate of 85.26%. There is no true/false positive rate for this category due to missing data during the test. Figure 9 shows a few instances of the field tests where the model was able to correctly detect the excavator and also a few instances where the model confused the excavator with other categories (i.e., truck, cable plough). The field testing indicated that the AI needs more data coming from the sensors to recalibrate it as the images captured by the device present some optical and chromatic distortions. This explains the high rate of errors.

## 6. AIoT Solution Deployment: SEA Gas Use-Case

SEA Gas was established in 2002 to own and operate the 700 km long underground high-pressure natural gas transmission pipeline system that delivers gas from Port Campbell in Victoria to Adelaide in South Australia. Since 2002, SEA Gas’ assets have expanded to include the Mortlake Pipeline in Victoria and lateral pipeline extensions. The PCA currently delivers approximately 40% of South Australia’s gas demand. South Australia relies heavily on natural gas for power generation, with gas-fired generation supplying nearly 50% of power generation in the state during financial year 2021. Gas is also supplied to industrial and commercial customers and for residential use.

**Figure 10:**
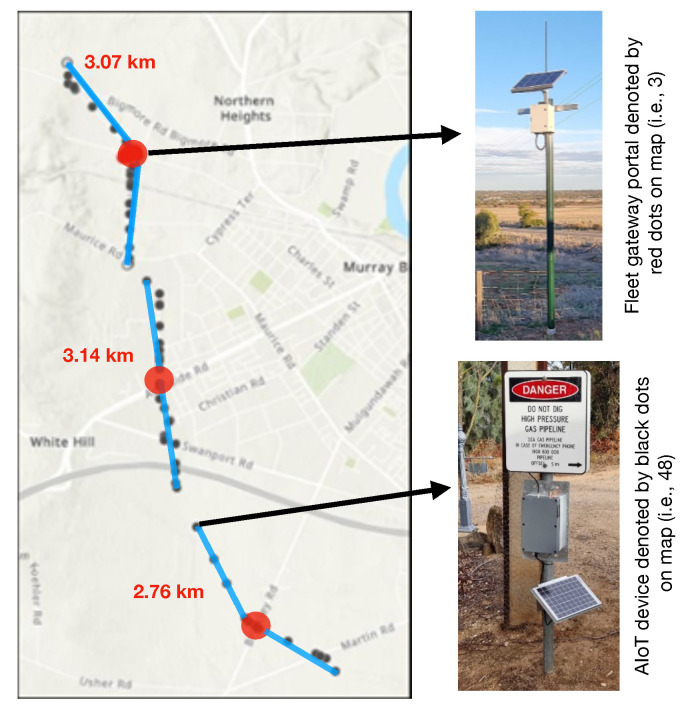
Location of the deployed AIoT devices for the SEA Gas use-case.

As part of this research, the proposed AIoT solution was deployed across the Murray Bridge section (approximately a 10 km stretch) of the pipeline operated by SEA Gas. The “Dial before you dig” markers along the pipeline easement were identified as good attachment points for the AIoT device’s deployment because of their distribution across the pipeline (i.e., coverage) and ground clearance for the camera sensor. In total, 48 marker posts were selected for the AIoT device’s deployment and were divided into three regions for Fleet Portal installments to ensure the line-of-sight communication (see Figure 10). The initial deployment of 48 devices allowed the project partners to assess several aspects of the solution, including power consumption, the robustness of the end-to-end communication network, and the performance of the AI model. This information will guide the future development of both the hardware and software components for improved performance.

## 7. Implication and Recommendations

The research aimed at enhancing industry threat-detection capabilities by introducing a continuous and intelligent monitoring of easement activity for the purpose of detecting potential threats typically involved in external interference incidents. The outcomes of the research include increasing awareness of threats and lowering the reaction time to prevent incidents for the infrastructure operator. While the current solution focuses on remote location relying on satellite communication to transmit alerts, the system can be adapted to take advantage of other wireless networks such as the conventional 4G. This will allow the deployment of the solution in risk hotspots such as areas under development in (sub)urban areas and road crossings. As one of the most significant risks to the pipeline industry, successful management of external interference will ensure the reliability and continuity of supply for operators and stakeholders, while also guaranteeing the safety of the community and the environment. The benefits of this research can be realized for pipelines both locally and globally and could potentially be broadened to other industries owning linear infrastructure, e.g., electricity transmission network service providers.

## 8. Conclusions

An AIoT smart device has been successfully developed and deployed in the field with the capability to detect an external interference threat to pipelines and alert the operator to avoid potential accidents. Two object-detection models (i.e., YOLOv4, DINO) were trained using the custom-developed Pipe-VisTA dataset to detect the threat from 13 categories. The proposed solution aimed to capture the image using a camera, detect the threat using the trained object-detection model, transmit the threat alert to the Fleet Portal using LoRaWAN, and host the alert information via the satellite network on a dashboard accessible to the operator/manager. Among the trained models, DINO was able to achieve the best validation and test mAP of 0.671 and 0.712, respectively. The tractor (i.e., 0.829), boring_rig (i.e., 0.823), and auger (i.e., 0.822) threat categories were identified as the best detected, while clay_delver (i.e., 0.256) and bobcat (i.e., 0.392) were reported as the worst. However, given the computational constraint of the NVIDIA Jetson Nano hardware for deployment, the simpler architecture of YOLOv4 with TensorRT optimization support available was used. The YOLOv4 model was able to achieve a validation and test mAP of 0.631 and 0.618, respectively. The developed AIoT device was tested and validated in the field to ensure the hardware’s and software’s functionality. The fully tested solution was deployed for a SEA Gas use-case site of 10 kilometers along the Murray Bridge section. In total, 48 AIoT devices and three Fleet Portals were deployed to cover the pipeline in three regions ensuring the line-of-sight communication from the devices to the portals. In the future, the performance of the AI model can be further improved by adding more images from the field in collaboration with pipeline companies covering a wide range of weather and lighting conditions. Furthermore, the camera system can be improved in terms of lens/image quality and power consumption. The power optimization will give longer battery times, which will enable tracking the duration of the threat in field. The dataset imbalance problem can be addressed using advanced synthetic-data-generation techniques including Generative AI models and the NVIDIA *Omniverse* platform.

## Figures and Tables

**Table 1:** Pipe-VisTA dataset classes.

auger	person	boring_rig	bobcat
excavator	tractor	ditch_witch	post_driver
cable_plough	ditch_witch_drill	clay_delver	truck
car			

**Table 2:** Specifications of the Leopard Imaging NANO-H136 camera.

**Focal Length**	1.58 mm	Pixel Size	1.12 μm × 1.12 μm
FOV	136 Degree	Operating Temperature	−20 °C–+60 °C
Active Pixels	3280 (H) × 2464 (V)	Operating Voltage	3 V
Dimensions (LWH)	150 mm × 25 mm × 15.3 mm		

**Table 3:** Model training parameters.

Parameter	YOLOv4	DINO
Backbone	CSPDarkNet53	Fan Tiny
Training Epochs	1000	200
Batch Size	8	4
Base Learning Rate	1e^−4^	2e^−4^
Number of Classes	13	13
Optimizer	Adam	–

**Table 4:** Validation and test performance of trained detection models for external interference threat detection.

Category	YOLOv4			DINO	
	Validation mAP	Test mAP		Validation mAP	Test mAP
auger	0.777	0.808		0.822	0.839
bobcat	0.669	0.642		0.392	0.260
boring_rig	0.710	0.746		0.823	0.851
cable_plough	0.639	0.632		0.716	0.749
car	0.549	0.532		0.672	0.675
clay_delver	0.169	0.197		0.256	0.331
ditch_witch	0.772	0.700		0.754	0.925
ditch_witch_drill	0.892	0.735		0.758	0.920
excavator	0.600	0.649		0.694	0.900
person	0.523	0.531		0.691	0.676
post_driver	0.644	0.598		0.684	0.626
tractor	0.767	0.762		0.829	0.903
truck	0.492	0.507		0.628	0.571
**Mean**	**0.631**	**0.618**		**0.671**	**0.712**

## Data Availability

Data are contained within the article.

## Abbreviations

The following abbreviations are used in this manuscript:

SMS	Safety Management Study
AIoT	Artificial Intelligence of Things
CNN	convolutional neural network
YOLO	You Only Look Once
SORT	Simple Online Real-time Tracking
ACID	Alberta Construction Image Dataset
DINO	DETR with Improved deNoising anchOr boxes
Pipe-VisTA	Pipeline Visual Threat Assessment
LPWAN	Low-Power Wide-Area
CSP	Cross-Stage Partial
SPP	Spatial Pyramid Pooling
MSE	mean-squared error
NMS	Non-Maximal Suppression
IoU	Intersection over Union
*TAO*	*Train Adapt Optimize*
GPU	Graphical Processing Unit
mAP	Mean Average Precision

## References

[1] Vairo T., Pontiggia M., Fabiano B. (2021). Critical aspects of natural gas pipelines risk assessments. A case-study application on buried layout. Process Saf. Environ. Prot..

[2] Jo Y.D., Crowl D.A. (2008). Individual risk analysis of high-pressure natural gas pipelines. J. Loss Prev. Process Ind..

[3] Bariha N., Mishra I.M., Srivastava V.C. (2016). Hazard analysis of failure of natural gas and petroleum gas pipelines. J. Loss Prev. Process Ind..

[4] Jo Y.D., Ahn B.J. (2002). Analysis of hazard areas associated with high-pressure natural-gas pipelines. J. Loss Prev. Process Ind..

[5] Liang W., Hu J., Zhang L., Guo C., Lin W. (2012). Assessing and classifying risk of pipeline third-party interference based on fault tree and SOM. Eng. Appl. Artif. Intell..

[6] Li X., Zhang Y., Abbassi R., Yang M., Zhang R., Chen G. (2021). Dynamic probability assessment of urban natural gas pipeline accidents considering integrated external activities. J. Loss Prev. Process Ind..

[7] Hu J., Zhang L., Liang W., Guo C. (2012). Intelligent risk assessment for pipeline third-party interference. J. Press. Vessel. Technol..

[8] Adewumi R., Agbasi O., Sunday E., Robert U. (2023). An Industry Perception and Assessment of Oil and Gas Pipeline Third-Party Interference. J. Int. Environ. Appl. Sci..

[9] APGA (1987). AS 2885: The Standard for High Pressure Pipeline Systems. https://www.apga.org.au/2885-standard-high-pressure-pipeline-systems.

[10] Ariavie G.O., Oyekale J.O. (2015). Risk Assessment of Third-Party Damage Index for Gas Transmission Pipeline around a Suburb in Benin City, Nigeria. Int. J. Eng. Res. Afr..

[11] Beller M., Steinvoorte T., Vages S. (2018). Inspecting challenging pipelines. Aust. Pipeliner Off. Publ. Aust. Pipelines Gas Assoc..

[12] Maslen S. (2014). Learning to prevent disaster: An investigation into methods for building safety knowledge among new engineers to the Australian gas pipeline industry. Saf. Sci..

[13] Papadakis G.A. (1999). Major hazard pipelines: A comparative study of onshore transmission accidents. J. Loss Prev. Process Ind..

[14] Biezma M., Andrés M., Agudo D., Briz E. (2020). Most fatal oil & gas pipeline accidents through history: A lessons learned approach. Eng. Fail. Anal..

[15] Iqbal H., Tesfamariam S., Haider H., Sadiq R. (2017). Inspection and maintenance of oil & gas pipelines: A review of policies. Struct. Infrastruct. Eng..

[16] Brunetti A., Buongiorno D., Trotta G.F., Bevilacqua V. (2018). Computer vision and deep learning techniques for pedestrian detection and tracking: A survey. Neurocomputing.

[17] Mpouziotas D., Karvelis P., Tsoulos I., Stylios C. (2023). Automated Wildlife Bird Detection from Drone Footage Using Computer Vision Techniques. Appl. Sci..

[18] Iqbal U., Bin Riaz M.Z., Barthelemy J., Perez P. (2022). Quantification of visual blockage at culverts using deep learning based computer vision models. Urban Water J..

[19] Iqbal U., Barthelemy J., Perez P., Davies T. (2022). Edge-Computing Video Analytics Solution for Automated Plastic-Bag Contamination Detection: A Case from Remondis. Sensors.

[20] Kim H., Kim H., Hong Y.W., Byun H. (2018). Detecting construction equipment using a region-based fully convolutional network and transfer learning. J. Comput. Civ. Eng..

[21] Roberts D., Golparvar-Fard M. (2019). End-to-end vision-based detection, tracking and activity analysis of earthmoving equipment filmed at ground level. Autom. Constr..

[22] Xiao B., Kang S.C. (2021). Vision-based method integrating deep learning detection for tracking multiple construction machines. J. Comput. Civ. Eng..

[23] Xiao B., Kang S.C. (2021). Development of an image data set of construction machines for deep learning object detection. J. Comput. Civ. Eng..

[24] Labelbox Labelbox: The Leading Training Data Platform for Data Labeling. https://labelbox.com.

[25] Haxhibeqiri J., De Poorter E., Moerman I., Hoebeke J. (2018). A survey of LoRaWAN for IoT: From technology to application. Sensors.

[26] Bochkovskiy A., Wang C.Y., Liao H.Y.M. (2020). Yolov4: Optimal speed and accuracy of object detection. arXiv.

[27] Zhang H., Li F., Liu S., Zhang L., Su H., Zhu J., Ni L.M., Shum H.Y. (2022). Dino: Detr with improved denoising anchor boxes for end-to-end object detection. arXiv.

[28] MMDet DINO. https://github.com/open-mmlab/mmdetection/tree/main/configs/dino.

[29] Kuznetsova A., Rom H., Alldrin N., Uijlings J., Krasin I., Pont-Tuset J., Kamali S., Popov S., Malloci M., Kolesnikov A. (2020). The open images dataset v4: Unified image classification, object detection, and visual relationship detection at scale. Int. J. Comput. Vis..

